# Antidepressant use and risk of intubation or death in hospitalized patients with COVID-19: A retrospective cohort study of clinical effectiveness

**DOI:** 10.3389/fpsyt.2022.951065

**Published:** 2022-09-15

**Authors:** Brian P. Brennan, Jiana Schnabel, Harrison G. Pope, James I. Hudson

**Affiliations:** ^1^Psychiatric Epidemiology Research Program and Biological Psychiatry Laboratory, McLean Hospital, Belmont, MA, United States; ^2^Department of Psychiatry, Harvard Medical School, Boston, MA, United States

**Keywords:** antidepressants, COVID-19, selective serotonin reuptake inhibitors, serotonin-norepinephrine reuptake inhibitors, retrospective cohort study, clinical effectiveness

## Abstract

Initial controlled trials of the serotonergic antidepressant fluvoxamine showed promise for treatment of mild to moderate COVID-19 in outpatients, although more recent outpatient data have been less encouraging. Turning to studies of hospitalized patients, a retrospective cohort study by Hoertel and associates in 2021 found a markedly reduced risk of intubation or death among patients hospitalized with COVID-19 who were receiving serotonergic antidepressants at the time of admission vs. those not receiving antidepressants. In an attempt to replicate these latter findings, we performed a similarly designed study of 500 individuals hospitalized with COVID-19 in a large academic hospital system who were taking a serotonergic antidepressant at the time of admission compared with two groups (*N* = 573 and *N* = 593) not receiving an antidepressant. In analyses controlling for demographic and clinical variables, we found no significant difference in effect between the antidepressant group and either of the two comparison groups [hazard ratios (95% CI) for intubation or death 1.1 (0.83–1.5) and 1.1 (0.86–1.5); and for death alone 1.3 (0.93–1.8) and 1.1 (0.85–1.7)]. Examining the results of our study, along with those of Hoertel et al. and three additional retrospective cohort studies in inpatients published in the interim, the data permit only very limited conclusions, with the findings on the effect of serotonergic antidepressants ranging from a strongly protective effect to no effect. Although there are numerous threats to validity that might account for this wide range of findings, we could not identify any principal factor or set of factors that could clearly explain the differences.

## Introduction

Growing evidence indicates that antidepressants, particularly selective serotonin reuptake inhibitors (SSRIs), may possess anti-inflammatory and antiviral properties (1–4)—suggesting that SSRIs might represent potential treatments for the novel coronavirus SARS-CoV-2 disease (COVID-19). A subset of SSRIs, specifically fluvoxamine and to a lesser degree fluoxetine and citalopram/escitalopram, are agonists at the sigma-1 receptor (5, 6), which has been identified as a potential target for COVID-19 therapeutics (7, 8). This property has been hypothesized to play a role in the efficacy of SSRIs in preventing severe COVID-19 (5, 6). Congruent with these findings, several initial studies of fluvoxamine in outpatients with COVID-19 (9–11), two of which were randomized placebo-controlled trials (9, 10), demonstrated a significant reduction in clinical deterioration on some outcome measures. However, more recent findings with fluvoxamine have been less encouraging: in April 2022, the U.S. Food and Drug Administration (FDA) declined a request for an emergency use authorization of fluvoxamine for outpatient treatment, citing two unpublished additional trials that had failed to demonstrate a benefit, both of which were terminated early for futility, and concluding that overall there were important “limitations in the available clinical study results” (available at https://www.accessdata.fda.gov/drugsatfda_docs/nda/2020/EUA%20110%20Fluvoxamine%20Decisional%20Memo_Redacted.pdf). Note that the FDA had access to extensive unpublished data derived both from recent studies that had appeared already in the literature and data from additional studies for which no data at all had been published thus far. Thus, the FDA report is an important source of information on outpatient studies of fluvoxamine in COVID, in that it contains substantial new information that has not yet appeared in the published scientific literature.

In addition to studies in outpatients, two inpatient studies have assessed the effect of open-label treatment of COVID-19 with fluvoxamine (12) and fluoxetine (13), respectively. Both reported a reduced risk of death in patients receiving these antidepressants compared with patients not receiving them, but these studies are severely limited by the lack of randomization and the absence of a placebo control group. Further details of these studies of antidepressants, with special attention to SSRIs (especially fluvoxamine), can be found in the recent comprehensive review by Hashimoto et al. (5). These authors also consider in depth the theoretical rationale for use of these medications, including a detailed discussion of potential mechanisms of action.

Given the urgency to develop novel COVID-19 treatments, additional data from retrospective cohort studies of individuals receiving antidepressants “incidentally” at the time of developing COVID-19 (that is, antidepressants prescribed for indications other than COVID-19) may help to assess clinical effectiveness. To address this issue, Hoertel et al. (14) assessed the association of antidepressant treatment with the primary outcome of risk of intubation or death among patients hospitalized with COVID-19 in 39 French hospitals early in the pandemic from January 24, 2020 to April 1, 2020. Comparing 345 patients prescribed antidepressants with patients not prescribed antidepressants, they found a markedly reduced risk of intubation or death [hazard ratio (95% confidence interval) = 0.52 (0.43–0.73), adjusted for age, sex, and clinical variables including co-occurring medical conditions] and risk of death alone [hazard ratio = 0.64 (0.48–0.86)]. The strongest evidence favored medications that inhibited serotonin reuptake (termed “serotonergic antidepressants” below in this paper), including SSRIs, serotonin-norepinephrine reuptake inhibitors (SNRIs), and mirtazapine.

After reading this paper, we attempted to replicate its findings for the case of serotoninergic antidepressants, using a design as similar as feasible to that of the original. Specifically, we performed a retrospective cohort study examining the risk of intubation or death among hospitalized COVID-19 patients treated in a large academic healthcare system who had either been prescribed a serotonergic antidepressant (“exposed”) or not prescribed a serotonergic antidepressant (“unexposed”) at the time of hospital admission.

Since the publication of Hoertel et al. study, and before our study was completed, three more similar retrospective cohort studies have appeared, with one study finding a strongly protective effect of antidepressants as a whole (15), another finding only a slight protective effect (16), and the third finding no protective effect at all (17). Before examining these three newer studies, we first present the results of our own study below and then discuss possible explanations for the disparate findings among all five of the retrospective cohort studies.

Note that additional studies pertaining the use of antidepressants in the inpatient setting have been published [e.g., Fei et al. (18) and Vai et al. (19); these have been reviewed by Hashimoto et al. (5)]. However, due to marked differences in design, the results of these studies cannot be directly compared to each other, or to the results of the present study, or to the results of the four other retrospective cohort studies that have appeared. Moreover, these studies are difficult to interpret due to inherent limitations of their designs, and hence provide only limited evidence bearing on the efficacy of antidepressants in treating severe COVID.

## Materials and methods

### Patient medical records

Prior to initiating study procedures, we obtained approval from the Mass General Brigham Institutional Review Board including a Waiver of Informed Consent/Authorization. Using a retrospective cohort design, we searched electronic medical records of all patients hospitalized in the Mass General Brigham Healthcare System (Massachusetts, USA) whose first (index) admission with a diagnosis of COVID-19 occurred between February 1, 2020 and March 3, 2021. The exposed group (antidepressant group; *N* = 500) represented all patients receiving treatment at the index admission with any of the of serotonergic antidepressants found by Hoertel et al. (14) to be associated with a reduced risk of intubation or death (SSRIs, SNRIs, and mirtazapine). There were two unexposed groups, each representing a random sample of patients with COVID-19 who were not receiving any antidepressant at the index admission. Non-Antidepressant Group 1 (*N* = 573) represented a sample frequency matched for age, sex, and race/ethnicity to patients in the antidepressant group and Non-Antidepressant Group 2 (*N* = 593) represented a sample frequency-matched for the same demographic characteristics as non-antidepressant group 1, and additionally frequency-matched for “comparative health,” as described below.

Data were obtained through the Mass General Brigham Healthcare System Research Patient Data Registry (RPDR), a centralized data registry that gathers clinical information from several Mass General Brigham hospitals (Massachusetts General Hospital, Brigham and Womens Hospital, Newton-Wellesley Hospital, Faulkner Hospital, and Mass General Brigham Salem Hospital). The RPDR has been described elsewhere (20) and has been used in several prior publications [e.g., (21–23)]. We located potential cases using the RPDR Query Tool, which can identify patients with specific demographics, diagnoses, laboratory tests, medications, molecular medicine, health history, microbiology, procedures, providers, and/or transfusion services.

We created an RPDR query for patients aged 18 years or older whose first admission to a Mass General Brigham hospital with a diagnosis of COVID-19 occurred between February 1, 2020 and March 3, 2021, and who were taking an antidepressant. This query identified 1,111 patients, whose data were then reviewed to confirm diagnosis and antidepressant status. Patients not taking an antidepressant within 48 h of admission were excluded. Additionally, any patients taking an antidepressant that was not included in the set of serotonergic antidepressants examined by Hoertel et al. (14) (i.e., fluoxetine, fluvoxamine, sertraline, paroxetine, citalopram, escitalopram, venlafaxine, duloxetine, desvenlafaxine, mirtazapine) were excluded. This review yielded a final sample of 500 patients, which we term the “Antidepressant” group.

We then submitted a second and third RPDR query for patients whose first admission to a Mass General Brigham hospital with a diagnosis of COVID-19 occurred during the same time interval, but who were not taking any antidepressant at the time of that admission. In the second query, we sought patients who were frequency-matched for age, sex, and race/ethnicity to patients in the antidepressant-exposed COVID-19 group. This query returned 1,003 patients. This group was similarly reviewed to confirm diagnosis and medication status. Any patients who were taking an antidepressant within the year prior to their index admission were excluded, resulting in a final unexposed group of 573 patients in Non-Antidepressant Group 1. In the third query, we sought patients who were frequency-matched for the same characteristics as for the second query, but who were also matched for “comparative health,” which is a proxy for healthcare utilization derived from the number of diagnostic codes, medication orders, and test results from hospital visits for each patient (23, 24). This query returned 1,007 patients, who were reviewed in the same manner as the previous groups, resulting in a final unexposed group of 593 patients in Non-Antidepressant Group 2. Note that of the patients from the second and third queries included in the final sample, there were 108 duplicates; that is, patients who were included in both groups.

### Data analysis

The observation period for all patients started with the day of the index admission and continued through the discharge date of the last hospital admission in the record up to a maximum of 118 days (which was the maximum observation time used by Hoertel et al.).

The primary outcome was the composite endpoint occurrence of either intubation or death (i.e., intubation without death, intubation followed by death, or death without intubation), and the secondary outcome was death (with or without intubation). An ancillary outcome was intubation (with or without death).

The primary measure of effect was the estimated hazard ratio from a proportional hazards model. The secondary measure of effect was the risk ratio. The models for the hazard ratio and risk ratio were adjusted, using inverse probability weighting (25), for age in categories (19–29, 30–39; 40–49; 50–59; 60–69; 70–79; 80–89; 90–103 y); race/ethnicity (using categories shown in Table 1); co-occurring disorders in nine ICD-10 diagnostic categories based on the index admission note (see Table 1); and secular time period (in four 90-day categories starting from the first admission date in the sample, which was March 12, 2020). For the proportional hazards models, we found no evidence for violations of the proportional hazards assumption through inspection of loglog plots, comparison of proportional hazards model-predicted curves with observed survival curves, and tests of weighted residuals.

**Table 1:** Demographic characteristics, co-occurring disorders, and frequency of outcomes in study groups.

	**Study group**		
	**Antidepressant^a^**	**Non-antidepressant 1^b^**	**Non-antidepressant 2^c^**
	***n*** **= 500**	***n*** **= 573**	***n*** **= 593**
**Demographic characteristics**			
Age, median (IQR) y	71 (60, 82)	71 (58, 80)	71 (59, 80)
**Sex**			
Female, No. (%)	284 (56.8)	297 (51.8)	316 (53.3)
Male, No. (%)	216 (43.2)	276 (48.2)	277 (46.7)
**Race**			
White, No. (%)	390 (78.0)	397 (69.3)	413 (69.7)
African American, No. (%)	45 (9.0)	57 (10.0)	54 (9.1)
Asian American or Pacific Islander, No. (%)	9 (1.8)	13 (2.3)	22 (3.7)
Other or not recorded, No. (%)	56 (11.2)	106 (18.4)	104 (17.5)
**Ethnicity**			
Non-Hispanic, No. (%)	413 (82.6)	435 (75.9)	460 (77.6)
Hispanic, No. (%)	71 (14.2)	109 (19.0)	108 (18.2)
Other or not recorded, No. (%)	16 (3.1)	29 (5.0)	25 (4.2)
**Co-occurring disorders (ICD-10 code)**			
Neoplasms (C00-D49)	46 (9.2)	90 (15.7)	81 (13.7)
Disorders of the blood and immune system (D50-D89)	123 (24.6)	164 (28.6)	185 (31.2)
Diabetes (E10, E11)	121 (24.2)	131 (22.9)	140 (23.6)
Obesity (E66)	57 (11.4)	68 (11.9)	47 (7.9)
Diseases of the circulatory system (I00-I99)	361 (72.2)	418 (73.0)	430 (72.51)
Diseases of the respiratory system (J00-J99)	335 (67.0)	404 (71.5)	421 (71.0)
Mood and anxiety disorders (F30-F48)	195 (39.0)	79 (13.8)	91 (15.4)
Other psychiatric disorders (F00-F29, F50-F99)	130 (26.0)	87 (15.2)	114 (19.2)
Diseases of the nervous system (G00-G99)	175 (35.0)	153 (26.7)	161 (19.2)
**Observation time**			
Days under observation, median (IQR)	7 (4, 15)	7 (4, 15)	7 (4, 16)
**Outcomes**			
Intubation or death composite, No. (%)	120 (24.0)	119 (20.7)	127 (21.4)
Death, No. (%)	101 (20.2)	87 (15.2)	102 (17.2)
Intubation	35 (7.0)	50 (8.7)	46 (7.8)

IQR, interquartile range.

a Patients prescribed one or more antidepressant medications on admission (see text and Table 2 for details).

b Frequency matched to antidepressant group by age, sex, and race/ethnicity.

c Frequency matched to antidepressant group by age, sex, race/ethnicity, and “comparative health.”

To evaluate the statistical significance of comparisons between groups, we adopted a hierarchical approach to control for type I error. We first evaluated the primary analysis of the primary outcome (the estimated hazard ratios for intubation or death of the Antidepressant Group vs. each of the two Non-Antidepressant Groups separately) with alpha set at 0.05, two-sided. If either of these tests yielded a *P*-value of <0.05, two-tailed, we considered the result statistically significant, and then proceeded to evaluate the statistical significance of the remaining comparisons using a false discovery rate (26) of 5%. If neither of the primary two comparisons was statistically significant, then we did not perform further formal testing, but rather reported point estimates along with nominal 95% confidence intervals. In such cases we also calculated the maximum hazard ratio that we could exclude (i.e., the maximum protective effect of antidepressants that is consistent with the data), using a test for non-equivalence, based on the 90% confidence interval of our measured effect sizes (27, 28). This test estimates a value of the hazard ratio for which there is <5% probability that the true hazard ratio exceeds this magnitude; that is, a 5% error rate.

In addition to the main analyses, we also performed exploratory subgroup analyses without formal statistical testing for each individual antidepressant, and for various classes of antidepressants, including SSRIs, SNRIs, mirtazapine, and sigma-1 receptor agonists [defined as escitalopram, citalopram, fluoxetine, or fluvoxamine, based on Table 1 in Hashimoto et al. (5)]. Also, because there is a potential effect of COVID vaccines on the outcomes of interest, and because we lacked reliable data on vaccine status, we performed a sensitivity analysis restricted to patients admitted prior to December 11, 2020, the date on which the first COVID vaccine became available in the United States

All analyses were performed using Stata 16.1 (Stata Corporation, College Station, Texas).

## Results

The frequency of demographic characteristics, co-occurring disorders, and study outcomes are presented in Table 1. The frequency of individual antidepressants and their median dose are presented in Table 2.

**Table 2:** Antidepressant medications prescribed to the 500 patients in the antidepressant group at the time of admission to the hospital, and hazard ratios for death in those prescribed individual antidepressants or classes of antidepressants vs. those not prescribed antidepressants.

			**Hazard ratio^c^** **(95% CI)**	
		**Dose, mg**	**vs. non-antidepressant**	**vs. non-antidepressant**
**Antidepressant medication**	**No.^a^ (%)**	**median (IQR)^b^**	**Group 1**	**Group 2**
**Selective serotonin reuptake inhibitors**				
Citalopram	61 (12.2)	20 (10, 20)	0.86 (0.39, 1.9)	0.77 (0.35, 1.7)
Escitalopram	47 (9.4)	10 (10, 20)	1.9 (0.99, 3.5)	1.7 (0.89, 3.1)
Fluoxetine	57 (11.4)	20 (20, 40)	0.94 (0.35, 2.5)	0.83 (0.31, 2.2)
Paroxetine	24 (4.8)	25 (20, 40)	0.57 (0.15, 2.2)	0.51 (0.13, 1.9)
Sertraline	127 (25.4)	50 (50, 100)	0.98 (0.59, 1.7)	0.87 (0.53, 1.4)
Any selective serotonin reuptake inhibitor	313 (62.6)		1.00 (0.67, 1.5)	0.89 (0.61, 1.3)
Sigma-1 receptor agonist (citalopram, escitalopram, or fluoxetine)	164 (32.8)		1.2 (0.69, 2.0)	1.03 (0.62, 1.7)
**Serotonin-norepinephrine reuptake inhibitors**				
Duloxetine	73 (14.6)	60 (30, 60)	1.1 (0.55, 2.3)	1.01 (0.49, 2.1)
Venlafaxine	39 (7.8)	75 (75, 150)	2.2 (1.1, 4.3)	1.9 (1.00, 3.8)
Any serotonin-norepinephrine reuptake inhibitor	111 (22.2)		1.6 (0.95, 2.7)	1.4 (0.86, 2.4)
Mirtazapine	128 (25.6)	14 (7.5, 15)	1.7 (1.1, 2.7)	1.5 (1.01, 2.4)

IQR, interquartile range.

a 54 patients were prescribed two antidepressants, and one patient was prescribed three antidepressants.

b Number of patients with missing dose information: citalopram (2); escitalopram (3); fluoxetine (2); paroxetine (2); sertraline (4); duloxetine (3); venlafaxine (2); mirtazapine (5).

c Estimate adjusted for age, sex, race, ethnicity, co-occurring disorders, and secular time period.

**Figure 1:**
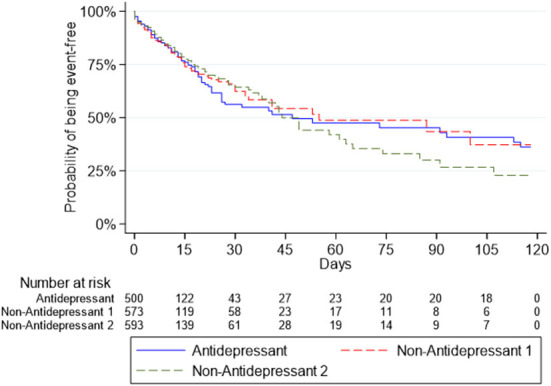
Survival curves for the composite outcome of intubation or death in the antidepressant group and the two non-antidepressant groups.

**Figure 2:**
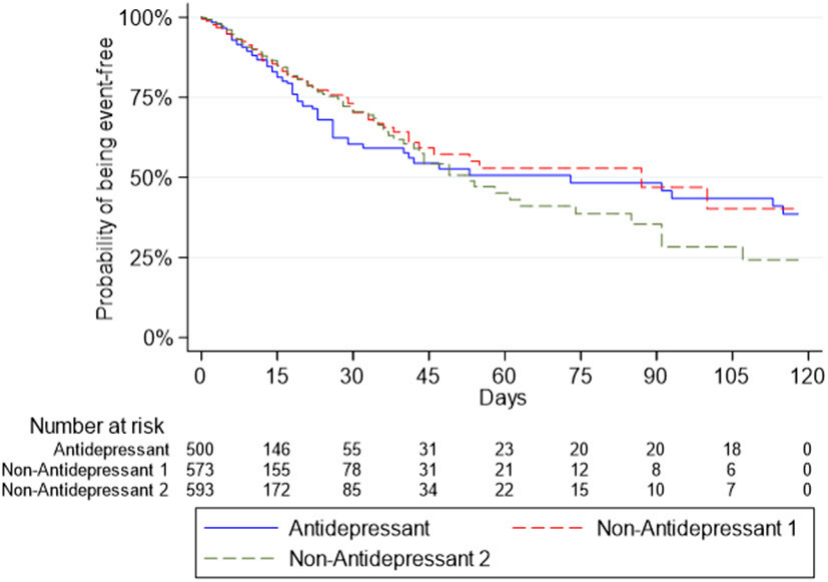
Survival curves for the outcome of death in the antidepressant group and the two non-antidepressant groups.

Survival curves for the Antidepressant Group and the two Non-Antidepressant Groups for death or intubation are presented in Figure 1, and for death in Figure 2.

The estimated hazard ratios and risk ratios for outcomes in the Antidepressant Group vs. the Non-Antidepressant Groups are presented in Table 3.

**Table 3:** Estimated hazard ratios and risk ratios for outcomes in antidepressant group vs. non-antidepressant group 1^a^ and non-antidepressant group 2^b^.

	**Outcome**		
**Between-group comparisons of risk**	**Death or intubation**	**Death**	**Intubation**
**Hazard ratio^c^ (95% CI)**			
Antidepressant vs. non-antidepressant 1	1.1 (0.83–1.5)	1.3 (0.93–1.8)	0.86 (0.53–1.4)
Antidepressant vs. non-antidepressant 2	1.1 (0.86–1.5)	1.1 (0.85–1.5)	1.1 (0.66–1.7)
**Risk ratio^c^ (95% CI)**			
Antidepressant vs. non-antidepressant 1	1.1 (0.86–1.4)	1.2 (0.92–1.7)	0.85 (0.53–1.3)
Antidepressant vs. non-antidepressant 2	1.1 (0.84–1.4)	1.1 (0.80–1.4)	1.0 (0.65–1.7)

CI, confidence interval.

a Frequency matched to antidepressant group by age, sex, and race/ethnicity.

b Frequency matched to antidepressant group by age, sex, race/ethnicity, and “comparative health.”

c Estimate adjusted for age, sex, race, ethnicity, co-occurring disorders, and secular time period.

Across all outcomes, the estimated hazard ratios and risk ratios were close to the null value of 1.0, with confidence intervals ranging from a modestly protective effect to a markedly harmful effect. Notably, the two primary comparisons—the hazard ratios for intubation or death in the Antidepressant Group vs. each of the Non-Antidepressant Groups—yielded results that were not statistically significant (*P* = 0.52 and *P* = 0.39, respectively), and thus we performed no further formal hypothesis testing as specified in the Methods. For the outcome of death or intubation, we could not exclude protective effects as great as a hazard ratio of 0.86 and harmful effects as great as a hazard ratio of 1.3; for the outcome of death, we could not exclude protective effects as great as a hazard ratio of 0.89 and harmful effects as great as a hazard ratio of 1.7.

In exploratory subgroup analyses of individual antidepressants and classes of antidepressants (including those identified as sigma-1 receptor agonists), we found no convincing evidence for differential effects, in that the hazard ratios were overlapping for all comparisons (see Table 2 for the estimated hazard ratios for the outcome of death; see Supplementary Table S1 for the full set of estimated hazard ratios and risk ratios for all outcomes). In our sensitivity analysis restricted to the period before COVID vaccines were available (comprising 68.2% of the total sample), the estimates changed slightly in the direction of the null value of 1.0 for hazard ratios and risk ratios, compared with those in the full sample (see Supplementary Table S1).

## Discussion

We sought to replicate the results of a retrospective cohort study of hospitalized patients with COVID-19 (14), which found that a range of serotonergic antidepressant medications (SSRIs, SNRIs, and mirtazapine) was associated with a marked protective effect for the joint of outcome of intubation or death, as well as for death alone. We failed to replicate these results, with hazard ratios and risk ratios little different from the null value. Notably, our study had sufficient power to exclude even a modest protective effect, namely a hazard ratio of <0.86 for intubation or death, and <0.89 for death alone.

Three additional retrospective cohort studies have now appeared, similar in design to those of Hoertel et al. and ours, finding markedly different estimates of the treatment effect of antidepressants in hospitalized patients with COVID-19. Oskotsky et al. (16) examined medical records from patients who presented with COVID-19 at 87 health care centers across the United States, including urgent care, observation, emergency, or inpatient settings, from January to September 2020. These investigators found a slightly reduced risk for death in the 3,401 patients prescribed SSRI antidepressants compared with those not prescribed SSRIs [risk ratio = 0.92 (0.85–0.99), adjusted for age, sex, race/ethnicity, encounter type, and clinical characteristics including co-occurring medications]. In an analysis restricted to individuals prescribed fluoxetine, they found that fluoxetine was associated with a moderately reduced risk [risk ratio = 0.72 (0.54–0.97)].

Rauchman et al. (17) examined medical records of patients hospitalized with COVID-19 at six hospitals in western United States between March 20, 2020 to March 20, 2021. These investigators found no significant difference in risk for death in the 832 patients prescribed SSRIs or SNRIs at admission compared with those who were not prescribed these antidepressants [odds ratio = 0.96 (0.79–1.2), adjusted for age, sex, and race/ethnicity, but not for clinical characteristics such as co-occurring medical conditions]. No subgroup analyses for individual antidepressants were reported.

Diez-Quevedo et al. (15) examined medical records from patients in Badalona, Spain hospitalized with COVID-19 from March 1, 2020 to November 20, 2020 and followed until December 17, 2020. They found a significantly reduced risk of death among the 164 patients who had been prescribed antidepressants during the year prior to admission, of whom only 118 were apparently taking antidepressants at the time of hospital admission [hazard ratio = 0.43 (0.25, 0.74)]. This study is difficult to interpret or to compare with the others considered above, because the details of exposure to antidepressants are unclear and the statistical analysis controlled for medical complications during the hospital admission itself—which could have been influenced by exposure to antidepressants.

Comparing all five retrospective cohort studies in hospitalized patients, the main challenge is to account for the striking differences in the magnitude of effects. For example, if the hazard ratios for mortality of 0.64 and 0.43 found by Hoertel et al. and Diez-Cuevedo et al., respectively, represent unbiased measures of the therapeutic effect, then antidepressants would be substantially superior to dexamethasone, the only therapeutic agent thus far demonstrated in randomized trials to significantly reduce mortality in severe COVID-19, with a risk ratio of 0.83 (0.75–0.93) reported in the largest and most rigorous clinical trial of this medication (29). By contrast, the three other observational studies found either a modest protective effect (Oskofsky et al.) or no effect at all (Rauchman et al. and ours) for serotonergic antidepressants as a whole. Thus, the therapeutic effect of antidepressants reported in observational studies of inpatients to date ranges from no effect to an effect surpassing any other known therapeutic agent.

**Figure 3:**
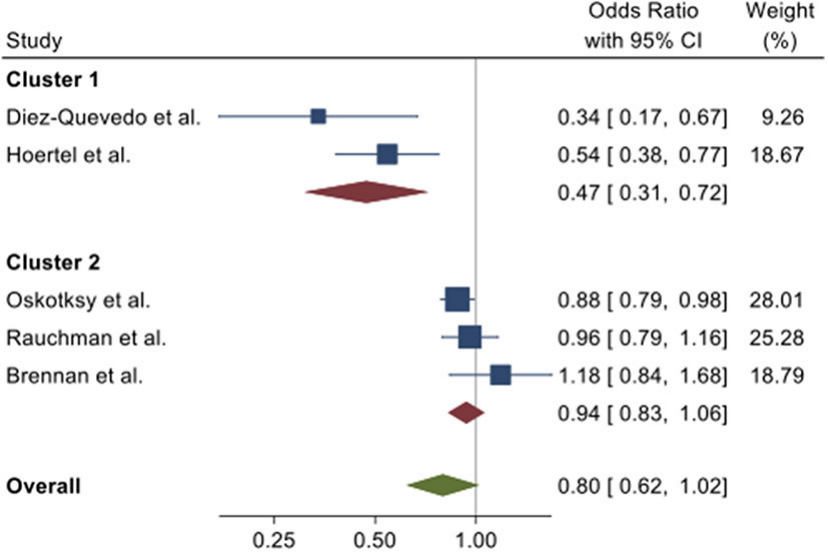
Forest plot of odds ratios with 95% confidence intervals from five observational studies for the odds for death in individuals prescribed antidepressants vs. those who were not prescribed antidepressants at the time of hospitalization for COVID-19. Combined effects were calculated using a random effects model (34). Tests for homogeneity: overall, Q(4) = 18.3, *P* < 0.001; cluster 1, Q(1) = 1.47, *P* = 023; cluster 2, Q(2) = 2.79, *P* = 0.25.

To illustrate this range of effect with respect to death as an outcome, we calculated odds ratios for all of the studies for the outcome of death, because each study provided information sufficient to obtain this measure, either directly in the paper or through reported data that allowed for its calculation. As shown in Figure 3, two clusters emerge. For the five studies together, there is strong evidence against a homogeneous effect, whereas within each cluster, the data are consistent with a homogeneous effect. In other words, the *within-cluster* differences in estimates are consistent with the role of chance, but the *between-cluster* estimates are not.

In attempting to explain these disparate findings, it is useful to first consider how well these various studies emulate an ideal randomized clinical trial, sometimes termed the “target trial” (25, 30). Several recent observational studies have exemplified how to emulate a target trial effectively (30–32). In our case, a rough sketch of the underlying target trial would be (a) a randomized controlled trial with a placebo arm and multiple active treatment arms (one for each antidepressant of interest), (b) enrolling individuals without COVID-19 from a given source population, who (c) would be followed until they developed a positive test for SARS-CoV-2, and then (d) would be treated using a uniform standard treatment algorithm that would exclude any non-study psychotropic medications. Those developing illness severe enough to warrant hospital-level care would then enter a second phase of the study, wherein the time of onset of clinical outcomes of interest (e.g., ventilatory assistance; death; remission of illness) would be recorded and then used to estimate measures of relative effect between treatment groups (e.g., hazard ratio, risk ratio).

Clearly, the available retrospective cohort studies fall well short of the rigor required for this target trial. Potential threats to the internal validity of the studies (i.e., factors that might bias *within-study* estimates of treatment effects) would include selection bias, bias due to measurement error, and unmeasured confounding. Additionally, even assuming perfect internal validity, a study might still lack external validity with respect to other source populations of interest, including those underlying the other observational studies. Finally, for all within-study and between-study comparisons, the effects of sampling variability (and more generally, the role of chance) must be considered.

All of the available studies are potentially vulnerable to selection bias (i.e., the study participants were not representative of individuals with severe COVID-19 in the underlying source population) and measurement error (e.g., inaccurate or missing data in the medical records reviewed, especially with regard to antidepressant use). We have little ability to estimate the degree of selection bias in the studies, nor what direction that bias might take. It is also difficult to assess the effect of measurement error, but this would be likely non-differential, thus tending to bias results toward the null.

All of the studies attempted to control for confounding by adjusting for demographic features (e.g., age and sex), and all but one (Rauchman et al.) adjusted for co-occurring medical conditions. Although the studies differed somewhat in their chosen set of co-occurring conditions and how these conditions were assessed, it is not clear that any of these choices was appreciably superior or inferior to the others in terms of controlling for confounding. Also important in pharmacoepidemiology studies is the threat of “confounding by indication,” where the indication for selecting a treatment also has an effect on the outcome (25, 33). In response to this issue, all of the studies except for Rauchman et al. controlled for indications for treatment (e.g., mood disorder). Moreover, in our study, the results of controlling for co-occurring conditions, including potential indications for antidepressant treatment, did not have any appreciable effect on the estimates. Thus, there is little reason to suspect that residual confounding by indication accounts for more than a small portion of the differences between the studies. Finally, although the statistical methods to control for confounding differed somewhat—including inverse-probability weighting (Hoertel et al. and our study), propensity scores (Oskotsky et al.), and inclusion of covariates into regression models (Rauchman et al. and Diez-Quevedo et al.)—all of these methods represent commonly used and valid choices, and thus would likely account for only a negligible portion of the differences in estimates between studies (25).

Looking next at between-study differences not attributable to bias, we first consider geographic and temporal differences. The five studies were done in France, Spain, and three settings in the United States, with the European studies yielding substantially higher estimates of treatment effect—conceivably reflecting differences in the characteristics of individuals prescribed antidepressants or differences in treatments administered for severe COVID-19.

The studies also differed in the time period examined, with the study of Hoertel et al. uniquely focusing only on the earliest weeks of the pandemic, whereas the other studies used later-occurring and longer time periods of collection. Thus, secular variations in the prevalence of SARS-CoV-2 variants, together with treatment advances as the pandemic progressed, might contribute to between-study differences. Notably, the mortality rate among the non-antidepressant groups in the Hoertel et al. study was 34%, whereas in the other, later studies it ranged from 16 to 23%.

Looking next at exposure to specific antidepressants, the distribution and dosage of antidepressants differed only modestly among the three studies reporting these data (Hoertel et al., Oskotsky et al. and our study), with the great majority of participants prescribed antidepressants in the recommended range for treatment of major depressive disorder. The study by Diez-Quevedo et al. provides the least amount of information on exposure and is difficult to interpret because nearly one third of participants were not receiving antidepressants at the time of hospitalization, but had received antidepressants only at some time in the previous year. Nevertheless, somewhat surprisingly, the study reported the greatest protective effect of antidepressants against death among the five studies. On balance, with the possible exception of the Diez-Quevedo et al. study, differences in distribution and dosage of antidepressants seem unlikely to have contributed substantially to the between-group differences in estimates of effects.

Looking at differences between the studies in demographic and clinical characteristics of participants, it seems unlikely that these differences would have had a major effect. The one possible exception is the Oskotsky et al. study, where the mean age of patients was 64 years—substantially lower than the three other studies reporting this information (mean 74 years for Hoertel et al.; median >71 years for Rauchman et al.; and median 74 years for ours). This lower age, coupled with the fact that 26% of the first encounters for COVID-19 were not at a hospital admission, but rather at a lower level of care, suggests that patients may have had a better prognosis and less severe illness than those in the other studies.

Finally, we consider the role of chance, with a focus on control for the effects of multiple comparisons. For the main comparisons between individuals prescribed vs. not prescribed *any* antidepressant (which was the primary comparison for all studies except Diez-Quevedo et al.), control for multiplicity would not seem to be required for valid interpretation. Here, the chance of falsely rejecting the null hypothesis is much <5% for Hoertel et al. and Diez-Quevedo et al., slightly <5% for Oskotsky et al., and much higher than 5% for Rauchman et al. and for our study.

Looking next at the “subgroup” analyses assessing the effect of individual antidepressants, Hoertel et al. report findings from ten individual antidepressants (examining only the primary analysis of the primary outcome), and obtain *P*-values of <0.05 for five of them. However, since this analysis involves ten individual comparisons, these *P*-values, uncorrected for multiple comparisons, would not meet the threshold for control of either the experiment-wise type I error rate or the false discovery rate at <5% using any of three standard statistical procedures (see Supplementary material). Oskotsky et al. report that their analytic plan called for subgroup comparisons of fluoxetine and fluvoxamine. But since only two individuals in their sample were prescribed fluvoxamine, they could not perform a comparison separately for this medication. The *P*-value for the fluoxetine-alone comparison was 0.03. Strictly speaking, however, this comparison should be considered as a part of a set of four study comparisons (Table 3 of their paper), and thus the experiment-wise type I error rate and false discovery rate would be >5% for the fluoxetine comparison. Thus, after correcting for the error rate inflation due to multiple comparisons, neither study provides convincing evidence that any *individual* antidepressant is protective against COVID-19 beyond a difference consistent with chance. These results are consistent with our own subgroup analyses, which yielded no evidence for differential effects by individual antidepressant, type of antidepressant (SSRI vs. SNRI vs. mirtazapine), or sigma-1 receptor agonism (high vs. low). We also found little change in our results when restricted to the time period before vaccines for COVID-19 were available.

In summary, although initial controlled trials of the serotonergic antidepressant fluvoxamine showed promise for treatment of mild to moderate COVID-19 in the outpatient setting, more recent data have produced more disappointing results, as noted above and as reviewed comprehensively by Hashimoto et al. (5). Turning to the inpatient setting, the five existing retrospective cohort studies of serotonergic antidepressant medications in inpatients with COVID-19 permit only very limited conclusions. The data remain insufficient to conclude that any individual antidepressant exhibits a statistically significant protective effect, and the studies' findings on the effect of serotonergic antidepressants as a whole range from a strongly protective effect to no effect. Although there are numerous threats to study validity that might account for this wide range of findings, we could not identify any principal factor or set of factors that could clearly account for the differences. Further observational studies, crafted to approach more closely the ideal “target trial” described above, may help to resolve these questions.

## Data availability statement

The data analyzed in this study is subject to the following licenses/restrictions: Privacy and confidentiality restrictions related to medical records from hospitalized patients at a large academic medical center. Requests to access these datasets should be directed to BB, bbrennan@partners.org.

## Ethics statement

The studies involving human participants were reviewed and approved by Mass General Brigham Institutional Review Board, Boston Massachusetts. Written informed consent for participation was not required for this study in accordance with the national legislation and the institutional requirements.

## Author contributions

BB participated in the conception and design of the study, the acquisition, analysis, and interpretation of the data, and writing and revising the manuscript. HP participated in the conception and design of the study, the interpretation of the data, and writing and revising the manuscript. JS participated in the acquisition and analysis of the data and revising the manuscript. JH participated in the conception and design of the study, the analysis and interpretation of the data, and writing and revising the manuscript. All authors contributed to the article and approved the submitted version.

## Conflict of interest

Author BB has received consulting fees from Rugen Therapeutics and Nobilis Therapeutics; and has received research grant support from Eli Lilly, Transcept Pharmaceuticals, and Biohaven Pharmaceuticals. Author JH has received consulting fees from Idorsia, Otsuka, and Sunovion; and has received research grant support from Boehringer-Ingelheim, Idorsia, and Sunovion. The remaining authors declare that the research was conducted in the absence of any commercial or financial relationships that could be construed as a potential conflict of interest.

## Publisher's note

All claims expressed in this article are solely those of the authors and do not necessarily represent those of their affiliated organizations, or those of the publisher, the editors and the reviewers. Any product that may be evaluated in this article, or claim that may be made by its manufacturer, is not guaranteed or endorsed by the publisher.

## References

[1] Carpinteiro A Edwards MJ Hoffmann M Kochs G Gripp B Weigang S. Pharmacological inhibition of acid sphingomyelinase prevents uptake of SARS-CoV-2 by epithelial cells. Cell Rep Med. (2020) 1:100142. 10.1016/j.xcrm.2020.100142 33163980 PMC7598530

[2] Schloer S Brunotte L Goretzko J Mecate-Zambrano A Korthals N Gerke V. Targeting the endolysosomal host-SARS-CoV-2 interface by clinically licensed functional inhibitors of acid sphingomyelinase (FIASMA) including the antidepressant fluoxetine. Emerg Microbes Infect. (2020) 9:2245–55. 10.1080/22221751.2020.1829082 32975484 PMC7594754

[3] Schloer S Brunotte L Mecate-Zambrano A Zheng S Tang J Ludwig S. Drug synergy of combinatory treatment with remdesivir and the repurposed drugs fluoxetine and itraconazole effectively impairs SARS-CoV-2 infection *in vitro*. Br J Pharmacol. (2021) 178:2339–50. 10.1111/bph.15418 33825201 PMC8251190

[4] Zimniak M Kirschner L Hilpert H Geiger N Danov O Oberwinkler H. The serotonin reuptake inhibitor Fluoxetine inhibits SARS-CoV-2 in human lung tissue. Sci Rep. (2021) 11:5890. 10.1038/s41598-021-85049-0 33723270 PMC7961020

[5] Hashimoto Y Suzuki T Hashimoto K. Mechanisms of action of fluvoxamine for COVID-19: a historical review. Mol Psychiatry. (2022) 27:1898–907. 10.1038/s41380-021-01432-3 34997196 PMC8739627

[6] Hashimoto Y Suzuki T Hashimoto K. Old drug fluvoxamine, new hope for COVID-19. Eur Arch Psychiatry Clin Neurosci. (2022) 272:161–3. 10.1007/s00406-021-01326-z 34476589 PMC8412866

[7] Gordon DE Hiatt J Bouhaddou M Rezelj VV Ulferts S Braberg H. Comparative host-coronavirus protein interaction networks reveal pan-viral disease mechanisms. Science. (2020) 370:abe9403. 10.1126/science.abe9403 33060197 PMC7808408

[8] Gordon DE Jang GM Bouhaddou M Xu J Obernier K White KM. A SARS-CoV-2 protein interaction map reveals targets for drug repurposing. Nature. (2020) 583:459–68. 10.1038/s41586-020-2286-9 32353859 PMC7431030

[9] Lenze EJ Mattar C Zorumski CF Stevens A Schweiger J Nicol GE. Fluvoxamine vs placebo and clinical deterioration in outpatients with symptomatic COVID-19: a randomized clinical trial. JAMA. (2020) 324:2292–300. 10.1001/jama.2020.22760 33180097 PMC7662481

[10] Reis G Dos Santos Moreira-Silva EA Silva DCM Thabane L Milagres AC Ferreira TS. Effect of early treatment with fluvoxamine on risk of emergency care and hospitalisation among patients with COVID-19: the TOGETHER randomised, platform clinical trial. Lancet Glob Health. (2022) 10:e42–51. 10.1016/S2214-109X(21)00448-4 34717820 PMC8550952

[11] Seftel D Boulware DR. Prospective cohort of fluvoxamine for early treatment of coronavirus disease 19. Open Forum Infect Dis. (2021) 8:ofab050. 10.1093/ofid/ofab050 33623808 PMC7888564

[12] Calusic M Marcec R Luksa L Jurkovic I Kovac N Mihaljevic S. Safety and efficacy of fluvoxamine in COVID-19 ICU patients: an open label, prospective cohort trial with matched controls. Br J Clin Pharmacol. (2021) 88:2065–73. 10.1111/bcp.15126 34719789 PMC8653355

[13] Nemeth ZK Szucs A Vitrai J Juhasz D Nemeth JP Hollo A. Fluoxetine use is associated with improved survival of patients with COVID-19 pneumonia: a retrospective case-control study. Ideggyogy Sz. (2021) 74:389–96. 10.18071/isz.74.0389 34856085

[14] Hoertel N Sanchez-Rico M Vernet R Beeker N Jannot AS Neuraz A. Association between antidepressant use and reduced risk of intubation or death in hospitalized patients with COVID-19: results from an observational study. Mol Psychiatry. (2021) 26:5199–212. 10.1038/s41380-021-01021-4 33536545

[15] Diez-Quevedo C Iglesias-Gonzalez M Giralt-Lopez M Rangil T Sanagustin D Moreira M. Mental disorders, psychopharmacological treatments, and mortality in 2150 COVID-19 Spanish inpatients. Acta Psychiatr Scand. (2021) 143:526–34. 10.1111/acps.13304 33792912 PMC8250711

[16] Oskotsky T Maric I Tang A Oskotsky B Wong RJ Aghaeepour N. Mortality risk among patients with COVID-19 prescribed selective serotonin reuptake inhibitor antidepressants. JAMA Netw Open. (2021) 4:e2133090. 10.1001/jamanetworkopen.2021.33090 34779847 PMC8593759

[17] Rauchman SH Mendelson SG Rauchman C Kasselman LJ Pinkhasov A Reiss AB. Ongoing use of SSRIs does not alter outcome in hospitalized COVID-19 patients: a retrospective analysis. J Clin Med. (2021) 11:70. 10.3390/jcm11010070 35011811 PMC8745642

[18] Fei L Santarelli G D'Anna G Moretti S Mirossi G Patti A. Can SSRI/SNRI antidepressants decrease the ‘cytokine storm' in the course of COVID-19 pneumonia? Panminerva Med. (2021). 10.23736/S0031-0808.21.04436-0. [Epub ahead of print]. 34240839

[19] Vai B Mazza MG Delli Colli C Foiselle M Allen B Benedetti F. Mental disorders and risk of COVID-19-related mortality, hospitalisation, and intensive care unit admission: a systematic review and meta-analysis. Lancet Psychiatry. (2021) 8:797–812. 10.1016/S2215-0366(21)00232-7 34274033 PMC8285121

[20] Nalichowski R Keogh D Chueh HC Murphy SN. Calculating the benefits of a research patient data repository. In: AMIA Annu Symp Proc. Chicago, IL (2006). 1044 p. PMC1839563 17238663

[21] Biederman J Fried R DiSalvo M Woodworth KY Biederman I Driscoll H. Further evidence of low adherence to stimulant treatment in adult ADHD: an electronic medical record study examining timely renewal of a stimulant prescription. Psychopharmacology. (2020) 237:2835–43. 10.1007/s00213-020-05576-y 32591937

[22] Orlova Y Rizzoli P Loder E. Association of coprescription of triptan antimigraine drugs and selective serotonin reuptake inhibitor or selective norepinephrine reuptake inhibitor antidepressants with serotonin syndrome. JAMA Neurol. (2018) 75:566–72. 10.1001/jamaneurol.2017.5144 29482205 PMC5885255

[23] Zhong QY Karlson EW Gelaye B Finan S Avillach P Smoller JW. Screening pregnant women for suicidal behavior in electronic medical records: diagnostic codes vs. clinical notes processed by natural language processing. BMC Med Inform Decis Mak. (2018) 18:30. 10.1186/s12911-018-0617-7 29843698 PMC5975502

[24] Murphy SN Weber G Mendis M Gainer V Chueh HC Churchill S. Serving the enterprise and beyond with informatics for integrating biology and the bedside (i2b2). J Am Med Inform Assoc. (2010) 17:124–30. 10.1136/jamia.2009.000893 20190053 PMC3000779

[25] Hernán MJR. Causal Inference: What If. Boca Raton, FL: Chapman & Hall/CRC (2020). Available online at: http://www.hsph.harvard.edu/miguel-hernan/causal-inference-book/ (accessed August 29, 2022).

[26] Benjamini Y Hochberg Y. Controlling the false discovery rate: a practical and powerful approach to multiple testing. J R Stat Soc Ser B. (1995) 57:289–300. 10.1111/j.2517-6161.1995.tb02031.x

[27] Berger RL Hsu JC. Bioequivalence trials, intersection-union tests and equivalence confidence sets. Stat Sci. (1996) 11:283–302. 10.1214/ss/1032280304

[28] Schuirmann DJ. A comparison of the 2 one-sided tests procedure and the power approach for assessing the equivalence of average bioavailability. J Pharmacokinet Biopharm. (1987) 15:657–80. 10.1007/BF01068419 3450848

[29] Recovery Collaborative Group. Dexamethasone in hospitalized patients with Covid-19. N Engl J Med. (2021) 384:693–704. 10.1056/NEJMoa2021436 32678530 PMC7383595

[30] Hernán MA Robins JM. Using big data to emulate a target trial when a randomized trial is not available. Am J Epidemiol. (2016) 183:758–64. 10.1093/aje/kwv254 26994063 PMC4832051

[31] Barda N Dagan N Cohen C Hernan MA Lipsitch M Kohane IS. Effectiveness of a third dose of the BNT162b2 mRNA COVID-19 vaccine for preventing severe outcomes in Israel: an observational study. Lancet. (2021) 398:2093–100. 10.1016/S0140-6736(21)02249-2 34756184 PMC8555967

[32] Dagan N Barda N Kepten E Miron O Perchik S Katz MA. BNT162b2 mRNA Covid-19 vaccine in a nationwide mass vaccination setting. N Engl J Med. (2021) 384:1412–23. 10.1056/NEJMoa2101765 33626250 PMC7944975

[33] Kyriacou DN Lewis RJ. Confounding by indication in clinical research. JAMA. (2016) 316:1818–9. 10.1001/jama.2016.16435 27802529

[34] DerSimonian R Laird N. Meta-analysis in clinical trials. Control Clin Trials. (1986) 7:177–8. 10.1016/0197-2456(86)90046-2 3802833

