# Supplementary material for: Antidepressant use and risk of intubation or death in hospitalized patients with COVID-19: A retrospective cohort study of clinical effectiveness
Source: Front Psychiatry. 2022 Sep 15;13:951065. doi: 10.3389/fpsyt.2022.951065 (PMC9520125; doi:10.3389/fpsyt.2022.951065)
Supplement: Supplementary file 1 [file Data_Sheet_1.pdf]

Brennan BP, Schnabel J, Pope HG Jr, Hudson JI. Antidepressant use and risk of intubation or death in hospitalized patients with COVID-19: a retrospective cohort study of clinical effectiveness. *Frontiers in Psychiatry* 2022.

Supplementary Material

1. Supplementary Data

Analyses of Individual Antidepressants and Classes of Antidepressants

The results of exploratory subgroup analyses of individual antidepressants and classes of antidepressants estimating the hazard ratio and risk ratio for between-group comparisons for individual antidepressants on the composite outcome of intubation or death and the outcome of death alone are presented in Table S1.

Correction for Multiple Comparisons for Subgroup Analyses of Individual Antidepressants Reported by Hoertel et al.

Overall considerations

As described in the main text of the paper, Hoertel et al. (1) present results of subgroup analyses for the between-group comparisons of outcomes of interest for each of 10 individual antidepressants. To determine whether the results of these analyses are consistent with the role of chance, control for error inflation due to multiple comparisons is required. Accordingly, we apply here three of most widely used procedures to the results of this study.

Broadly, there are three standard classes of methods (2, 3). The first, and most traditional, method is to control the *experimental-wise type I error rate* through an adjustment of *P*-values, such as by using the data-independent Bonferroni procedure or the data-driven Holm procedure (4). The second, which is gaining in popularity for many applications, including biomedical studies involving a large number of comparisons (5), is to control the *false discovery rate*, such as by using the data-driven Benjamini-Hochberg procedure (6). The third is to use a *hierarchical testing* procedure, the most common of which is based on a pre-specified ordered set of hypotheses.

In our analysis, we employed three procedures: 1) control for experiment-wise error rate using the Bonferroni procedure; 2) control for the experiment-wise error rate using the Holm procedure; and 3) control for the false discovery rate using Benjamini-Hochberg procedure. We note that for the third class of methods, the hierarchical testing procedures, there is no rational way to apply this method because there was no *a priori* reason to hypothesize that any individual antidepressant would be more efficacious than any other – with the exception of fluvoxamine, which could not be evaluated because only one individual in the sample was taking this medication.

We confine our analysis to the primary analysis (described in the abstract as a “multivariate-Cox model with inverse probability weighting” (1)) for the ten individual antidepressants reported in Table 2 of the paper. However, similar conclusions would be reached if one considered the entire set of comparisons reported, which include the primary analysis of various classes of antidepressants, along with secondary outcomes and secondary analyses pertaining to all of the antidepressant subgroups.

Control for experiment-wise error rate using the Bonferroni procedure

The Bonferroni adjustment for the *P*-value is α divided by the number of comparisons. With $\alpha = 0.05$, we have a corrected α of 0.05/10 = 0.005. Since all *P*-values in are higher than this threshold, none of the comparisons is statistically significant (Table S2).

Control for experiment-wise error rate using the Holm procedure

The Holm procedure begins by evaluating the lowest P-value at a threshold of α/m, with m being the total number of comparisons. If the lowest observed *P*-value is less than α / m, then it is considered statistically significant, and the procedure continues. However, if the lowest observed *P*-value is not less than α / m, then the procedure stops and no comparison is considered statistically significant. The threshold for the first comparison is the same as that for the Bonferroni procedure, which is 0.005. Thus, when applied to the data from Hoertel et al., since all P-values are >0.005, the procedure stops at the first step and none of the comparisons is statistically significant (Table S2).

Control for the false discovery rate using the Benjamini-Hochberg

The Benjamini-Hochberg procedure takes the observed *P*-values, $P_{(k)}$ (where k goes from 1 to m, with $m = 10$ in this case), in order from lowest to highest and compares them with respect to a list of ordered thresholds, which are calculated as $\alpha*k/m$. The level of significance corresponds to the highest for k, such that $P_{(k)} < \alpha*k/m$. In this case, we compare the values of $P_{(k)}$ to the threshold value of $\alpha*k/m = 0.05*k/10 = 0.005*k$. None of the observed $P_{(k)}$ is less than the corresponding threshold value of $0.005*k$, and therefore, none of the comparisons is statistically significant (Table S2).

Conclusions

Using any of three standard methods to control for multiple comparisons, we find that none of the ten subgroup comparisons can be considered statistically significant. Furthermore, since none of these comparisons even approached statistical significance after correction, it is likely that no alternative method of correction would find them significant at an acceptable experiment-wise type I error rate or false discovery rate. Thus, the differences reported cannot be considered inconsistent with the role of chance.

2. Supplementary Tables

Table S1. Estimated Hazard Ratios and Risk Ratios for Between-Group Comparisons on the Composite Outcome of Intubation or Death and the Outcome of Death Alone for Individual Antidepressants; Subgroups of Antidepressants; and Sensitivity Analysis of All Antidepressants for Time Period Before Availability of COVID Vaccines

	**Intubation or Death**				**Death**			
	**Hazard Ratio[a] (95% CI)**		**Risk Ratio[a] (95% CI)**		**Hazard Ratio[a] (95% CI)**		**Risk Ratio[a] (95% CI)**	
**Antidepressant Medication**	**Non-Antidepressant Group 1**	**Non-Antidepressant Group 2**	**Non-Antidepressant Group 1**	**Non-Antidepressant Group 2**	**Non-Antidepressant Group 1**	**Non-Antidepressant Group 2**	**Non-Antidepressant Group 1**	**Non-Antidepressant Group 2**
Selective Serotonin Reuptake Inhibitors								
Citalopram	0.69 (0.35, 1.4)	0.71 (0.36, 1.4)	0.55 (0.28, 1.1)	0.54 (0.27, 1.1)	0.86 (0.39, 1.9)	0.77 (0.35, 1.7)	0.60 (0.27, 1.3)	0.52 (0.23, 1.1)
Escitalopram	1.02 (0.55, 1.9)	1.05 (0.57, 1.9)	0.96 (0.48, 1.9)	0.93 (0.47, 1.9)	1.9 (0.99, 3.5)	1.7 (0.89, 3.1)	1.3 (0.6, 2.7)	1.1 (0.5, 2.3)
Fluoxetine	0.86 (0.37, 2.0)	0.88 (.38, 2.1)	0.74 (0.32, 1.7)	0.72 (0.32, 1.6)	0.94 (0.35, 2.5)	0.83 (0.31, 2.2)	0.67 (0.24, 1.8)	0.57 (0.21, 1.6)
Paroxetine	1.04 (0.33, 3.2)	1.1 (0.34, 3.3)	1.1 (0.43, 2.6)	0.92 (0.60, 1.4)	0.57 (0.15, 2.2)	0.51 (0.13, 1.9)	0.72 (0.23, 2.2)	0.62 (0.20, 1.9)
Sertraline	0.84 (0.51, 1.4)	0.86 (0.53, 1.4)	0.95 (0.62, 1.4)	0.92 (0.60, 1.4)	0.98 (0.59, 1.7)	0.87 (0.53, 1.4)	1.1 (0.69, 1.7)	0.94 (0.60, 1.5)
Any selective serotonin reuptake inhibitor	0.85 (0.59, 1.2)	0.88 (0.62, 1.2)	0.83 (0.59, 1.1)	0.80 (0.58, 1.1)	1.00 (0.67, 1.5)	0.89 (0.61, 1.3)	0.90 (0.62, 1.3)	0.77 (0.54, 1.1)
Sigma-1 receptor agonist (citalopram, escitalopram, or fluoxetine)	0.86 (0.54, 1.4)	0.89 (0.56, 1.4)	0.72 (0.46, 1.2)	0.70 (0.44, 1.1)	1.2 (0.69, 2.0)	1.03 (0.62, 1.7)	1.2 (0.67, 2.4)	1.1 (0.57, 2.0)

Serotonin-Norepinephrine Reuptake Inhibitors								
Duloxetine	0.83 (0.43, 1.6)	0.85 (0.44, 1.6)	0.98 (0.55, 1.8)	0.95 (0.53, 1.7)	1.1 (0.55, 2.3)	1.01 (0.49, 2.1)	1.3 (0.67, 2.4)	1.1 (0.57, 2.0)
Venlafaxine	2.4 (1.4, 4.1)	2.4 (1.4, 4.2)	2.4 (1.6, 3.7)	2.3 (1.6, 3.5)	2.2 (1.1, 4.3)	1.9 (1.00, 3.8)	2.5 (1.5, 4.2)	2.1 (1.3, 3.6)
Any serotonin-norepinephrine reuptake inhibitor	1.4 (0.91, 2.2)	1.5 (0.94, 2.3)	1.6 (1.1, 2.3)	1.6 (1.2, 2.3)	1.6 (0.95, 2.7)	1.4 (0.86, 2.4)	1.8 (1.1, 2.7)	1.5 (0.99, 2.3)
Mirtazapine	1.4 (0.94, 2.1)	1.4 (0.97, 2.1)	1.5 (1.05, 2.1)	1. (1.02, 2.0)	1.7 (1.1, 2.7)	1.5 (1.01, 2.4)	1.8 (1.2, 2.6)	1.5 (1.1, 2.)
All Antidepressants Before COVID Vaccines Available[b]	1.00 (0.72, 1.4)	0.94 (0.75, 1.3)	1.03 (0.78, 1.4)	0.95 (0.73, 1.2)	1.1 (0.79, 1.6)	0.96 (0.69, 1.3)	1.2 (0.83, 1.6)	0.96 (0.71, 1.3)

Abbreviation: CI - confidence interval

a- Estimate adjusted for age, sex, race, ethnicity, co-occuring disorders, and secular time period.

b- Restricted to 1,147 patients admitted prior to December 11, 2000 (68.4% of full sample of 1,666 patients)

Table S2. Control for Multiple Comparisons for Subgroup Analyses of Individual Antidepressants Reported by Hoertel et al. (2021)

			**Bonferroni**		**Holm**		**Benjamini-Hochberg**	
**Comparison Number**	**Antidepressant**	**Observed *P*-value**[a]	**Threshold Value**	**Statistically Significant?**[b]	**Threshold Value**	**Statistically Significant?**[c]	**Threshold Value**	**Statistically Significant?**[d]
1	Paroxetine	0.026	0.005	No	0.005	No	0.005	No
2	Fluoxetine	0.034	0.005	No	N/A[e]	No	0.010	No
3	Venlafaxine	0.036	0.005	No	N/A[e]	No	0.015	No
4	Mirtazapine	0.040	0.005	No	N/A[e]	No	0.020	No
5	Escitalopram	0.046	0.005	No	N/A[e]	No	0.025	No
6	Mianserin	0.10	0.005	No	N/A[e]	No	0.030	No
7	Citalopram	0.32	0.005	No	N/A[e]	No	0.035	No
8	Duloxetine	0.59	0.005	No	N/A[e]	No	0.040	No
9	Sertraline	0.37	0.005	No	N/A[e]	No	0.045	No
10	Amitriptyline	0.96	0.005	No	N/A[e]	No	0.050	No

a- Listed by observed *P*-values, from lowest to highest

b- Is observed *P*-value less than threshold value?

c- Is observed *P*-value less than threshold value AND has procedure has not engaged stopping rule in any of the previous comparisons (see footnote e and details in text)?

d- Is there a value of *k* = comparison number, such that *P(k)* is less than the threshold value (see details in text)?

e- Procedure stops at first comparison, because the *P*-value for the first comparison is not less than the threshold value; thus, no further thresholds are calculated (see text).

References

1. Hoertel N, Sanchez-Rico M, Vernet R, Beeker N, Jannot AS, Neuraz A, et al. Association between antidepressant use and reduced risk of intubation or death in hospitalized patients with COVID-19: results from an observational study. Mol Psychiatry. 2021;26(9):5199-212.

2. Alosh M, Bretz F, Huque M. Advanced multiplicity adjustment methods in clinical trials. Stat Med. 2014;33(4):693-713.

3. Dmitrienko A, D'Agostino R, Sr. Traditional multiplicity adjustment methods in clinical trials. Stat Med. 2013;32(29):5172-218.

4. Holm S. A simple sequentially rejective multiple test procedure. Scand J Statistics. 1979;6:65-70.

5. Glickman ME, Rao SR, Schultz MR. False discovery rate control is a recommended alternative to Bonferroni-type adjustments in health studies. J Clin Epidemiol. 2014;67(8):850-7.

6. Benjamini Y, Hochberg Y. Controlling the false discovery rate: a practical and powerful approach to multiple testing. Journal of the Royal Statistical Society, Series B. 1995;57(1):289-300.
